# Triptolide induces apoptotic cell death of human cholangiocarcinoma cells through inhibition of myeloid cell leukemia-1

**DOI:** 10.1186/1471-2407-14-271

**Published:** 2014-04-17

**Authors:** Xiwei Ding, Bin Zhang, Qingshan Pei, Jianmei Pan, Shuling Huang, Yan Yang, Zhu Zhu, Ying Lv, Xiaoping Zou

**Affiliations:** 1 Department of Gastroenterology, Drum Tower Hospital, Affiliated to Medical School of Nanjing University, Nanjing, China; 2 Department of Gastroenterology, Renmin Hospital of Wuhan University, Wuhan, China; 3 Department of Gastroenterology, Jinan Central Hospital, Affiliated to Shandong University, Jinan, China

**Keywords:** Triptolide, Cholangiocarcinoma, Mcl-1, Apoptosis

## Abstract

**Background:**

Cholangiocarcinoma (CCA), a devastating neoplasm, is highly resistant to current chemotherapies. CCA cells frequently overexpress the antiapoptotic protein myeloid cell leukemia-1(Mcl-1), which is responsible for its extraordinary ability to evade cell death. Triptolide, a bioactive ingredient extracted from Chinese medicinal plant, has been shown to inhibit cell proliferation and induce apoptosis in several cancers.

**Methods:**

CCK-8 assay was performed to detect cell survival rate *in vitro*. DAPI staining and Flow cytometry were used to analyze apoptosis. Western blot was performed to determine the expression levels of caspase-3, caspase-7, caspase-9, PARP, and Mcl-1. Quantitative real-time PCR and immunofluorescence were used to detect the expression levels of Mcl-1. The nude mice xenograft model was used to evaluate the antitumor effect of triptolide *in vivo*.

**Results:**

Triptolide reduced cell viability in cholangiocarcinoma cell lines in a dose- and time-dependent manner, with IC_50_ values of 12.6 ± 0.6 nM, 20.5 ± 4.2 nM, and 18.5 ± 0.7 nM at 48 h for HuCCT1, QBC939, and FRH0201 respectively. Triptolide induced apoptosis in CCA cell lines in part through mitochondrial pathway. Using quantitative real-time PCR, western blot and immunofluorescence, we have shown that triptolide downregulates Mcl-1 mRNA and protein levels. Furthermore, triptolide inhibited the CCA growth *in vivo*.

**Conclusions:**

Triptolide has profound antitumor effect on CCA, probably by inducing apoptosis through inhibition of Mcl-1. Triptolide would be a promising therapeutic agent for CCA.

## Background

Cholangiocarcinoma (CCA) is a highly malignant adenocarcinoma arising from the bile duct epithelial cells. The overall incidence and mortality rates of this fatal neoplasm appear to be increasing in several western countries [1-5]. CCA is characterized by a poor prognosis and overall low survival rates [6]. Surgical resection and liver transplantation are considered the only two potentially curative therapies. Unfortunately, majority of patients have advanced disease at the time of diagnosis and miss the optimal time for these curative treatment options. Currently available chemotherapic agents and conventional radiotherapy are not effective in prolonging long-term survival of CCA patients. Therefore, it is necessary to develop novel effective therapeutic strategies against this neoplasm.

Natural products have played a significant role over the years in the development of anticancer drugs. Triptolide is a principal bioactive ingredient of the Chinese herb Tripterygium wilfordii Hook F, which has been used in traditional Chinese medicine for treating autoimmune and inflammatory diseases for centuries [7]. In addition to its immunosuppressive and anti-inflammatory properties, triptolide has attracted extensive research interest in its antitumor effects. Previous studies have shown that triptolide is highly effective against a variety of cancer types, including melanoma, breast cancer, bladder cancer, and gastric cancer [8,9]. However, little is known about its impact on CCA.

The aim of this study was to investigate the efficacy and possible mechanism of triptolide against human CCA both *in vitro* and in a nude mice xenograft model. We found that triptolide inhibited cell survival at low nanomolar concentrations. The mechanism by which triptolide induced CCA cell death was probably by inducing apoptosis through inhibition of myeloid cell leukemia-1 (Mcl-1). *In vivo* assay also verified the anticancer effect of triptolide in this neoplasm. Our findings provide the experimental basis for using triptolide against human CCA in the future.

## Methods

### Reagents

Triptolide and 4′, 6-diamidino-2-phenylindole (DAPI) were purchased from Sigma (St. Louis, MO, USA). Alexa Fluor 488 (green) conjugated goat anti-rabbit secondary antibody and TRIzol were purchased from Invitrogen (Carlsbad, CA, USA). Cell counting kit-8 (CCK-8) was provided by Dojindo Laboratories (Kumamoto, Japan). Annexin V Apoptosis Detection kit FITC was purchased from eBioscience (San Diego, CA, USA). PrimeScript™ RT Master Mix and SYBR Premix Ex Taq reagents were purchased from Takara Biotechnology (Dalian, China). Caspase-3 activity assay kit was obtained from Beyotime Institute of Biotechnology (Hangzhou, China). Antibodies against caspase-3 (9662), caspase-7 (9492), caspase-9 (9502), PARP (9532), Mcl-1(5453), and actin (4967) were from Cell Signal Technology (Boston, MA, USA). Antibody against Mcl-1(sc-819) was from Santa Cruz Biotechnology (San Jose, CA, USA). HRP-linked anti-rabbit secondary antibody was from Cell Signal Technology. Antibody against Ki67 (Kit-0005-2) was obtained from Maixin Bio (Fuzhou, China).

### Cell culture

The intrahepatic CCA cell line HuCCT1 was obtained from Jiangsu Province Hospital (Nanjing, China). The extrahepatic CCA cell line QBC939 was a generous gift from Dr. Qiang Huang (Anhui Provincial Hospital, Hefei, China). The extrahepatic CCA cell line FRH0201 was obtained from Qilu Hospital of Shandong University (Jinan, China). QBC939 and FRH0201 were cultured in RPMI-1640 medium supplemented with 10% FBS. HuCCT1 was cultured in DMEM containing 10% FBS. All cells were maintained in a humidified incubator at 37°C with 5% CO_2._

### Cell viability assay

Cell viability was detected by CCK-8 assay. Cells were seeded into 96-well plates at 3 × 10^3^ cells per well and cultured overnight at 37°C. After treatment with triptolide at indicated concentrations for 24 and 48 h, 10 uL of the tetrazolium substrate was added to each well of the plate. Plates were incubated at 37°C for 1 h, after which the absorbance at 450 nm was measured. All experiments were done in triplicate and repeated three independent times.

### DAPI staining for apoptosis assessment

Cells were seeded into 6-well plates and cultured as described above. After indicated treatments, the cells were stained with 10 μg/mL DAPI for 15 min. Morphologic changes in apoptotic nuclei were observed and photographed under the fluorescence microscope. Apoptosis was defined as the presence of nuclear condensation or fragmentation on DAPI staining.

### Caspase-3 activity assay

Caspase-3 activity was detected using a caspase-3 assay kit from Beyotime. Briefly, CCA cells were exposed to different concentrations of triptolide for 12 h. The cells were washed twice with PBS, and lysed for 15 min on ice. Lysates were centrifuged at 10,000 g for 15 min at 4°C. For each well in a 96-well microplate, cell lysate (10 μl), assay buffer (80 μl), and caspase-3 substrate (Ac-DEVD-pNA, 10 μl) were combined. The samples were incubated at 37°C for 12 h and their absorbance was recorded at 405 nm using a microplate reader (BioTek, USA).

### Annexin V-FITC apoptosis assay

Apoptosis induction by triptolide was assessed by flow cytometry using Annexin V-FITC labeling. About 2 × 10^5^ cells/well were seeded in a 6-well plate and treated with different concentrations of triptolide for 24 h. Apoptosis was determined using Annexin V-FITC Apoptosis Detection kit and performed according to the instructions. Data were analyzed using FlowJo Version 8.7.

### Quantitative real-time PCR

Total RNA was extracted from cultured cells using TRIzol reagent and cDNA was synthesized using PrimeScript™ RT Master Mix according to the manufacturer’s instructions. Quantitative real-time PCR experiments were done with the 7500 Real-time PCR System (Applied Biosystems) using SYBR Premix Ex Taq reagents. The primers for quantifying Mcl-1 are 5′-CATTTCTTTTGGTGCCTTTGTG-3′ and 5′-CCAGTCCCGTTTTGTCCTTAC-3′. The primers for quantifying β-actin are 5′-GGGCACGAAGGCTCATCATT-3′ and 5′-AGCGAGCATCCCCCAAAGTT-3′. All data were normalized to the human β-actin. All experiments were done in triplicate and repeated three independent times.

### Western blot

Cells were lysed in RIPA buffer (50 mM Tris–HCl with pH 7.4, 150 mM NaCl, 0.25% deoxycholic acid, 1% NP-40, 1 mM EDTA). The protein in cell lysates was resolved by 8–12% sodium dodecyl sulfate–polyacrylamide gel electrophoresis and transferred to polyvinylidene fluoride membranes. The membranes were blocked by 5% non-fat dry milk in Tris buffered saline containing 0.1% Tween-20 for 1 hour at room temperature. Then they were incubated with the desired primary antibodies (1:1000 dilutions) overnight, followed by appropriate HRP-conjugated secondary antibodies (1:2000 dilutions). Antibody binding was detected using Millipore Immobilon Western Chemiluminescent HRP Substrate according to the manufacturer’s instructions.

### Immunofluorescence

Cells were cultured on 24-well plates, fixed with 4% paraformaldehyde, and blocked for one hour with 5% normal goat serum, followed by incubation with polyclonal antibodies against Mcl-1 (1:50) overnight at 4°C. Cells were then rinsed with PBS and incubated with Alexa Fluor 488-conjugated goat anti-rabbit secondary antibody (1: 200). Cells were counter-stained with DAPI (2 μg/ml) and examined by fluorescence microscopy.

### Xenograft in nude mice

The mouse experiments were conducted in the Animal Laboratory Center of Nanjing Drum Tower Hospital (Nanjing, China). Nude mice were purchased from the Comparative Medicine Center of Yangzhou University (Yangzhou, China). The animal experiments were approved by the Institutional Animal Care and Use Committee of Nanjing Drum Tower Hospital, Nanjing University Medical School.

HuCCT1 cells (1 × 10^7^cells) were suspended in 100 μl serum free medium and injected subcutaneously into the left flank of 4- to 6-week old male BALB/c nu/nu nude mice. Tumor size was measured with digital caliper and calculated as V = LS^2^/2 (where L is the longest diameter and S is the shortest diameter). After 7 days when the tumor size reached about 150 mm^3^, mice were randomly segregated into two groups with five mice in each group. Mice were treated daily by intraperitoneal injection of triptolide at a dose of 0.2 mg/kg body weight or vehicle control. Tumor volume and animal weight were measured every three days and at the end of about 3 weeks after treatment, mice were sacrificed. Tumors were excised, weighted, fixed in 10% neutral formalin, and embedded in paraffin for subsequent histological analysis.

### Immunohistochemistry

Paraffin embedded sections were deparaffinized and rehydrated in graded alcohols and xylene using standard procedures, and either stained with H & E for histology, or Ki67 as a measure of cell proliferation. For Ki67 immunohistochemistry, sections were incubated with primary antibodies overnight at 4°C followed by secondary antibody for 1 h at room temperature. Slides were developed by DAB reagent and counter stained with heamtoxylin for 30 s.

### Statistical analysis

All the statistical analyses were performed using Prism 5.0 (Graphpad Software Inc., San Diego, USA). Data are expressed as the mean ± SEM of three independent experiments at least. The Student’s *t* test was used to compare the control and treated groups. In experiments involving more than three groups, one-way ANOVA and Bonferroni’s *post hoc* test were used. All statistically tests were two sided. Differences were considered statistically significant when the *P* value was less than 0.05.

## Results

### The effect of triptolide on CCA cell viability

**Figure 1:**
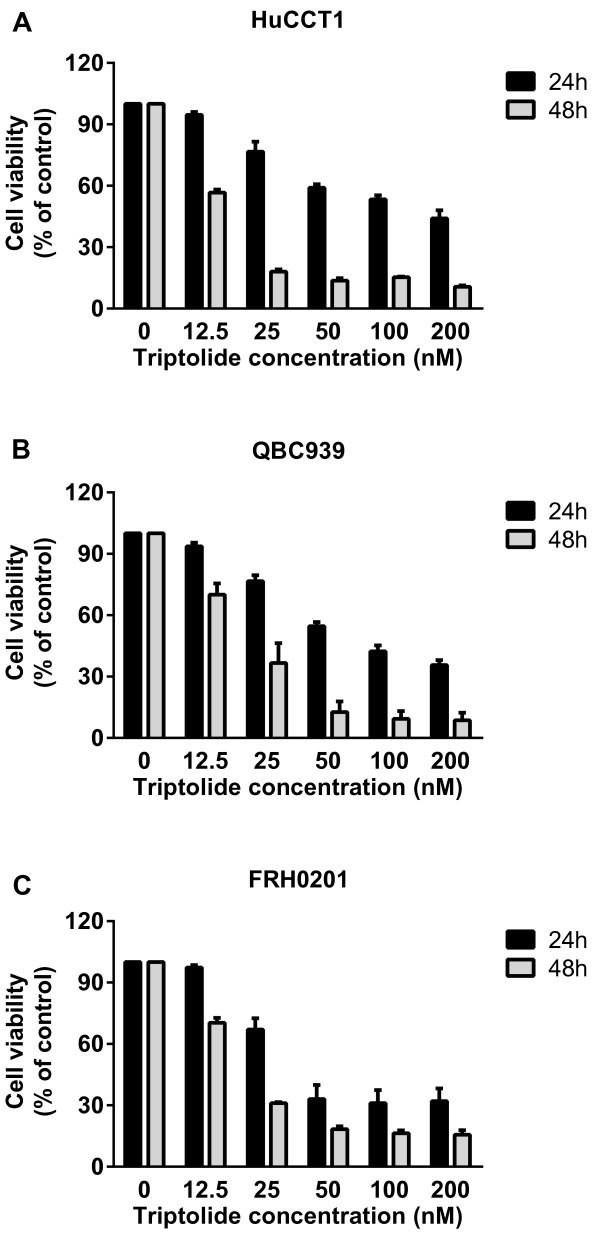
**Triptolide inhibited cell survival of CCA cells. (A-C)** HuCCT1 **(A)**, QBC939 **(B)**, and FRH0201 **(C)** cells were incubated with triptolide (0–200 nM) for 24 and 48 h. Cell viability was evaluated by CCK-8 assay.

We evaluated the effect of triptolide on three CCA cell lines: HuCCT1, QBC939, and FRH0201. Cell viability was assessed by CCK-8 assay after incubation in medium containing triptolide at concentrations of 0 to 200 nM for 24 and 48 h. All the cell lines tested showed a significant dose- and time-dependent decrease in cell viability after triptolide treatment (Figure 1). The levels of cytotoxicity were indicated as the concentration that inhibits the cell survival by 50%, IC_50_. As shown in Table 1, triptolide had an extremely low IC_50_ of nanomolar level on CCA cells. The IC_50_ values of triptolide on HuCCT1, QBC939, and FRH0201 at 48 h were 12.6 ± 0.6 nM, 20.5 ± 4.2 nM, and 18.5 ± 0.7 nM respectively.

**Table 1:** **IC**_**50 **_**for cholangiocarcinoma cell lines treated with triptolide**

**Cell lines**	**IC**_**50 **_**at 24 h (nM)**	**IC**_**50 **_**at 48 h (nM)**
HuCCT1	124.1 ± 21.5	12.6 ± 0.6
QBC939	82.2 ± 5.1	20.5 ± 4.2
FRH0201	54.1 ± 13.1	18.5 ± 0.7

Cells were plated in 96-well plates and treated with indicated concentrations (0-200 nM) of triptolide for 24 and 48 h. IC_50_ values were calculated using a nonlinear dose–response curve fit and expressed as mean ± SEM.

### Triptolide induces apoptosis in CCA cells

**Figure 2:**
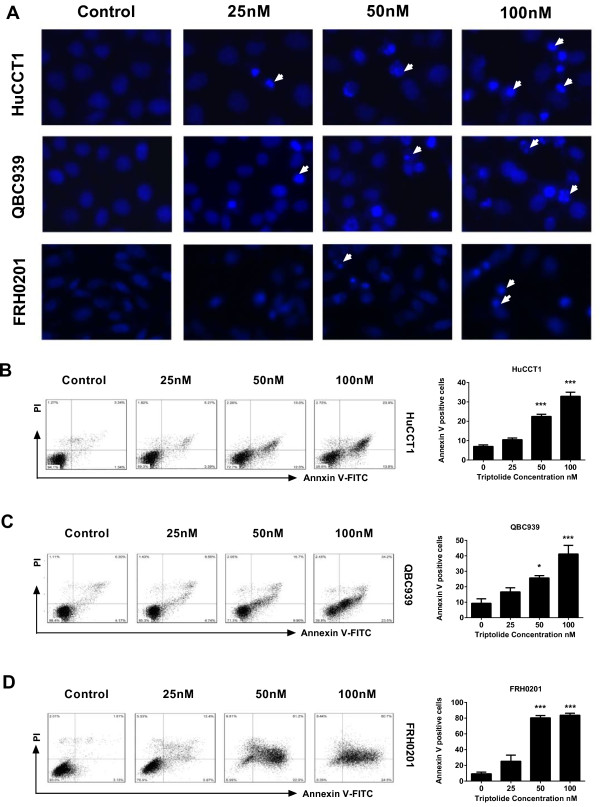
**Triptolide induced apoptosis of CCA cells. (A)** DAPI staining (original magnification, ×200) was used to determine the apoptosis. Arrows indicate cells with nuclear condensation and fragmentation. **(B-D)** HuCCT1 **(B)**, QBC939 **(C)**, and FRH0201 **(D)** cells were incubated with triptolide (0-100 nM) for 24 h and Annexin V levels were assessed by flow cytometry. *p < 0.05, ***p < 0.001, compared with control.

To elucidate the mechanism by which triptolide causes cell death in CCA cells, we used DAPI staining and Annexin V-FITC assay to evaluate whether triptolide could induce apoptosis in CCA cells. As shown in Figure 2A, nuclear condensation and fragmentation on DAPI staining was obvious in triptolide-treated cells. The induction of apoptosis by triptolide was validated by increased staining of Annexin V. Treatment of HuCCT1 cells with 50 nM to 100 nM triptolide for 24 h resulted in an approximately 3.2-fold to 4.7-fold increase in apoptosis (Figure 2B). Likewise, treatment of QBC939 and FRH0201 cells with triptolide shows a dose-dependent increase in Annexin V staining (Figure 2C and D). Our data show that treatment with triptolide induces apoptosis in all CCA cell lines tested.

### Triptolide induces caspase activation and PARP cleavage in CCA cells

**Figure 3:**
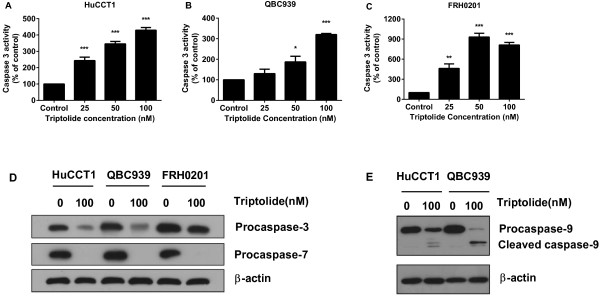
**Triptolide induced caspase activation in CCA cells. (A-C)** Caspase-3 activity in CCA cells were analyzed after treatment with indicated concentrations of triptolide for 12 h. Data are presented as fold increases as compared with control cells. **(D)** Protein expression of caspase-3 and caspase-7 were analyzed by western blot after treatment with triptolide (100 nM) for 24 h. **(E)** Protein expression of caspase-9 was analyzed by western blot after treatment with triptolide (100 nM) for 24 h. *p < 0.05, **p < 0.01, ***p < 0.001, compared with control.

Caspase-9 is known to be downstream of cytochrome c release from the mitochondria in the intrinsic apoptosis pathway and is commonly used as a measure of intrinsic apoptosis. As seen in Figure 3E, treatment with triptolide decreased procaspase-9 and increased cleaved caspase-9 in both HuCCT1 and QBC939 cells. Activated caspase-9 activates caspase-3 and caspase-7, which in turn induces PARP cleavage. As shown in Figure 3A-C, triptolide dose-dependently increased caspase 3 activity as early as 12 h after treatment. Triptolide treatment also reduced the procaspase-3 and procaspase-7 in all three CCA cell lines (Figure 3D). Our results suggest that triptolide induces apoptosis, at least in part, through the intrinsic mitochondrial pathway.

### The effect of triptolide on Mcl-1 levels in CCA cells

**Figure 4:**
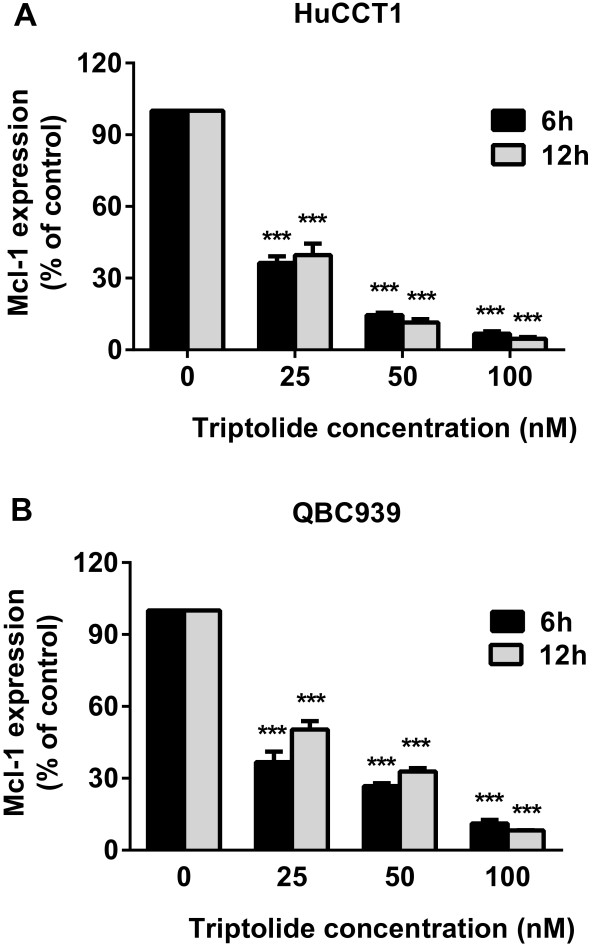
**Triptolide inhibited Mcl-1 mRNA expression in HuCCT1 and QBC939 cells. (A-B)** Triptolide (25–100 nM) significantly reduced Mcl-1 mRNA expression (as assessed by real-time PCR) in both HuCCT1 **(A)** and QBC939 **(B)** cells. Expression of Mcl-1 was normalized against the housekeeping gene β-actin. ***p < 0.001, compared with control.

**Figure 5:**
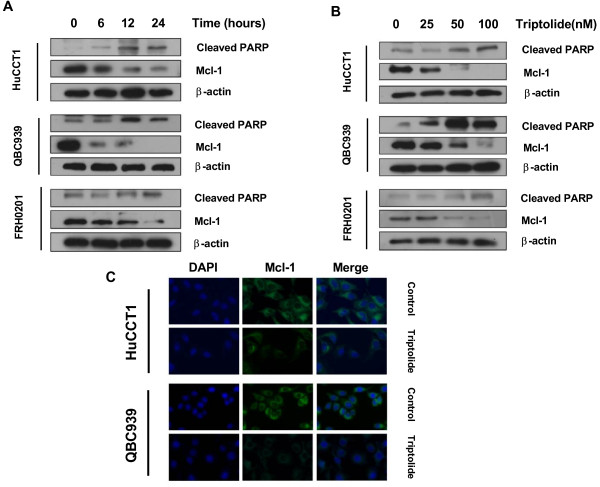
**Triptolide time- and dose-dependently inhibited Mcl-1 protein expression and induced PARP cleavage in CCA cells. (A)** Cells were treated with 100 nM triptolide for indicated times or **(B)** with indicated concentrations of triptolide for 12 h. Whole cell extracts were prepared and analyzed using indicated antibodies. β-actin served as a loading control. **(C)** HuCCT1 and QBC939 cells were treated with 100 nM triptolide for 24 h and then analyzed for the expression of Mcl-1 by immunofluorescence (original magnification, ×200).

Our next step was to investigate the mechanism by which triptolide induces apoptosis. CCA cells frequently overexpress the antiapoptotic Bcl-2 family member Mcl-1, which has been shown to play a critical role in cell survival and apoptosis resistance of CCA [10]. Instead, other Bcl-2 family members like Bcl-2 or Bcl-xL play less important roles in the apoptosis of CCA cells. We hypothesized that triptolide induced apoptosis in CCA cells by inhibiting Mcl-1, and then systematically tested our hypothesis. The effect of triptolide on Mcl-1 expression was analyzed by real-time PCR, western blot, and immunofluorescence. Our results clearly show that triptolide with concentrations of 25 nM to 100 nM significantly decreased Mcl-1 mRNA expression of HuCCT1 and QBC939 cells at both 6 and 12 h, as compared with control cells not exposed to triptolide (Figure 4). In accordance with its effect on mRNA expression, triptolide also significantly decreased Mcl-1 protein levels in all CCA cells tested in a time- and dose- dependent way (Figure 5A and B). The decrease of Mcl-1 protein levels was also corresponded with an increase of apoptosis as indicated by increase of cleaved-PARP, suggesting that Mcl-1 downregulation may be a primary mechanism for the proapoptosis property of this drug. Mcl-1-inhibiting effects of triptolide were further confirmed by immunofluorescence in HuCCT1 and QBC939 cells (Figure 5C). Taken together, these results show that triptolide causes CCA cell death via downregulation of Mcl-1 expression.

### The effect of triptolide on CCA tumor growth *in vivo*

**Figure 6:**
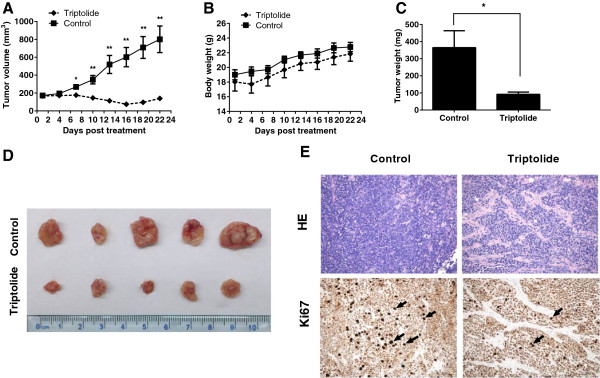
**Triptolide inhibited CCA cell growth *in vivo*.** HuCCT1 cells were injected subcutaneously into the left flanks of athymic nude mice. When tumors reached a size of approximately 150 mm^3^, mice were i.p. with triptolide (0.2 mg/kg) or vehicle control every day for a total of 22 days. **(A)** Tumor volume was measured every three days using calipers and calculated as described in Materials and Methods (n = 5). **(B)** The mouse weight was measured every three days to monitor the drug effects. **(C)** At the end of the study, the excised tumors from each group were weighed. **(D)** Resected tumors from each group were photographed. **(E)** H & E and Ki67 staining in the tumor tissues of triptolide and vehicle treated mice (original magnification, ×200). Arrows show the Ki67 expression in tumor tissue. *p < 0.05, **p < 0.01, compared with control.

The anticancer effect of triptolide was further analyzed in a xenograft tumor model by transplanting HuCCT1 cells into nude mice. On the 7th day after implantation, mice were randomly divided into 2 groups, with 5 tumor-bearing mice in each group. Intraperitoneal injections of triptolide (0.2 mg/kg/d) significantly inhibited the tumor growth for 22 days compared with the vehicle group (Figure 6A). Tumor weight was also significantly reduced after triptolide treatment (Figure 6C). Ki67 is a proliferation marker required for cell-cycle progression, replication, and DNA repair. As shown in Figure 6E, tumor cell proliferation, as indicated by Ki67, was reduced significantly by the treatment of triptolide. To assess the toxicity of the treatment, we measured mouse body weight every three 3 days and no significant weight loss was observed in the mice treated with triptolide compared with vehicle group during the whole treatment period (Figure 6B).

## Discussion

In this study, we explored the biological effects of a natural product, triptolide, on CCA cells. This drug induces CCA cell death with low nanomolar IC_50_ values. The mechanism by which triptolide induces CCA cell death is through induction of apoptosis via the inhibition of Mcl-1.

In the present study, incubation with triptolide significantly decreased CCA cell viability in three CCA cell lines: HuCCT1, QBC939, and FRH0201. Although the mechanism of triptolide against tumor cells has not been fully understood, it has been shown to induce apoptosis in some cancer cells [11-13]. Then we examined whether triptolide induced CCA cell death through the apoptosis pathway. Triptolide dose-dependently induced apoptosis in all CCA cell lines tested, with significant increases in Annexin V staining and nuclear condensation and fragmentation. Caspase activation is the key event of apoptosis. One pathway of caspase activation is the intrinsic mitochondrial pathway, from which cytochrome c translocates into the cytosol, which then interacts with apoptosis protease activator factor-1 and activates caspase-9. Activated caspase-9 then activates downstream caspase-3 and/or caspase-7, which in turn induces PARP cleavage. Our results show that, following incubation with triptolide, caspase-9 is activated, accompanied by significant increase in caspase-3 activity and PARP cleavage, thus strongly suggesting that triptolide induced cell death through the mitochondrial apoptotic pathway. These results concur with those reported in pancreatic cancer cell lines and leukemic cell lines treated with triptolide [12,13].

The Bcl-2 protein family comprises major regulators of cell survival which can promote or suppress apoptosis [14]. Mcl-1 is an antiapoptotic member of Bcl-2 protein family, which can promote cell survival through suppression of cytochrome c release from mitochondria [15]. Accumulated evidence indicates that Mcl-1 is essential for development and survival of acute myelogenous leukemia and various solid tumors including CCA [16,17]. Mcl-1 mediates the resistance to apoptosis by blocking the mitochondria pathway [18]. Mcl-1 is frequently overexpressed in CCA cells and human CCA tissues [10,19,20]. The elevated expression of this protein plays a pivotal role in protecting the CCA cells from apoptosis and promoting CCA cell survival [10,19,21]. Instead, other Bcl-2 family members like Bcl-2 and Bcl-xL has less important antiapoptotic effects in CCA [10]. The implication of these reports is that inhibiting Mcl-1 expression, using genetic or pharmacological approaches, could be a potentially effective strategy against this neoplasm. However, only a small number of pharmacologic Mcl-1 inhibitors have been identified [16]. Our study has shown that low concentrations of triptolide can inhibit both mRNA and protein expression of Mcl-1 as early as 6 h after the treatment and induce potent apoptosis through mitochondria pathway. To the best of our knowledge, this is the first report demonstrating Mcl-1 as a target of triptolide in CCA cells. However, how triptolide downregulates Mcl-1 transcription is not clear and needs further exploration.

We also examined the effect of triptolide on CCA growth using a nude mice model of CCA derived from the highly malignant CCA cell line HuCCT1. Daily intraperitoneal injection of 0.2 mg/kg triptolide substantially suppressed the growth of established CCA tumors, indicating the tumor regression potential of triptolide. Importantly, no toxicity was observed at the dose of 0.2 mg/kg per day. We show here for the first time that triptolide inhibits CCA growth in a nude mice xenograft model. Although triptolide is an effective antitumor compound *in vitro*, its use *in vivo* has been restricted owing to its low solubility in water. Recently, a water-soluble analog of triptolide, Minnelide, designed and synthesized by the University of Minnesota, has overcome this issue and shown considerable efficacy in preclinical studies against pancreatic cancer, osteosarcoma, and lung carcinoma [22-24]. In addition, Minnelide has shown to be more effective than gemcitabine in the preclinical study against pancreatic cancer. Minnelide is now undergoing Phase I clinical trial against the advanced gastrointestinal tumors and may represent a novel effective compound against cancer. Resistance to apoptosis is a key factor preventing response to conventional therapy in cancer. Triptolide has been shown to cooperate with several conventional chemotherapeutic drugs to induce apoptosis in different cancer cell lines and increase the sensitivity to various chemotherapeutic agents [25-29]. Whether triptolide can sensitize the CCA to conventional chemotherapy has not been investigated and requires further exploration because CCA is highly resistant to current chemotherapeutic agents. The cytokine tumor necrosis factor-related apoptosis-inducing ligand (TRAIL) is an attractive agent for the treatment of advanced cancers including CCA, through selective induction of cancer cell apoptosis. Prior work [30,31] has shown that triptolide may sensitize CCA cells to TRAIL via apoptosis. Taniai et al. [10] has described that many cholangiocarcinoma cells are resistant to TRAIL-mediated apoptosis through overexpression of Mcl-1. Our result shows that triptolide decreases Mcl-1 expression, suggesting that triptolide sensitizes cholangiocarcinoma cells to TRAIL partially through Mcl-1 inhibition. This combination therapy with triptolide and TRAIL has important therapeutic potential in clinical translation for cholangiocarcinoma.

## Conclusions

In summary, our study provides evidence that triptolide inhibits the tumor growth in CCA both *in vitro* and *in vivo*. The main mechanism of this effect might be inducing intrinsic apoptosis via inhibition of Mcl-1. These findings suggest that triptolide is a promising therapeutic agent for CCA.

## Abbreviations

CCA: Cholangiocarcinoma; Mcl-1: Myeloid cell leukemia-1; DAPI: 4′, 6-diamidino-2-phenylindole; CCK-8: Cell counting kit-8; PARP: Poly (ADP-ribose) polymerase; DAB: 3, 3′-diaminobenzidine tetrahydrochloride.

## Competing interests

The authors declare no competing interest.

## Authors’ contributions

YL and XZ conceived and supervised the study. XD, QP, and YY performed the experiments. XD, BZ, and ZZ analyzed and interpreted the data. XD and BZ drafted and revised the manuscript. JP and SH provided technical support. All authors read and approved the final manuscript.

## Pre-publication history

The pre-publication history for this paper can be accessed here:

http://www.biomedcentral.com/1471-2407/14/271/prepub
